# A cluster randomised controlled trial, process and economic evaluation of two large-scale quality improvement interventions embedded with a national clinical audit to improve the care for young adults with type 2 diabetes (EQUIPD2): study protocol

**DOI:** 10.1186/s13012-025-01479-8

**Published:** 2026-01-06

**Authors:** Michael Sykes, Bethan Copsey, Tracy Finch, Adam Martin, Alice Hankin, Melissa Girling, Elaine O’Halloran, Ruth Medcalf-Bell, Bryony Dawkins, Jenny McSharry, Eimear Morrissey, Shivani Misra, David Gable, Garry Tan, Alex Berry, Kayleigh Steele, Florence Day, Lauren Moreau, Rosemary Dewey, Robbie Foy

**Affiliations:** 1 Northumbria University, Newcastle upon Tyne, UK; 2 University of Leeds, Leeds, UK; 3 University of Galway, Galway, Ireland; 4 Imperial College London, London, UK; 5 Imperial College Healthcare NHS Foundation Trust, London, England; 6 National Diabetes Audit, London, UK; 7 Diabetes UK, London, UK; 8 Expert By Experience, London, UK; 9 NHS West Yorkshire Integrated Care Board, Wakefield, UK

**Keywords:** Audit and feedback, Quality improvement, Diabetes, Randomised controlled trial, Learning health system

## Abstract

**Background:**

Young adults (18–39 years) with type 2 diabetes have an increased loss of life expectancy and a greater risk of complications such as retinopathy, sexual health problems and foot disease than people diagnosed with type 2 diabetes later in life. Globally, there are increasing numbers of young adults with type 2 diabetes. Evidence describes both care (for example, prescribing) and improvement practices (for example, case management) that improve outcomes for people with type 2 diabetes.

The National Diabetes Audit (NDA) provides feedback describing variation in both care and outcomes in young adults. Feedback facilitation can increase the effectiveness of audit feedback. Working collaboratively between researchers, audit providers, patients, clinicians and policy-makers, we have developed two feedback facilitation interventions deliverable at scale across England. We will evaluate whether theory-informed virtual educational materials with email support (low-intensity intervention) and / or virtual workshops (medium-intensity intervention) improve outcomes for young adults with type 2 diabetes.

**Methods:**

An efficient, pragmatic cluster randomised controlled trial using routine data with a theory-informed process and economic evaluation. The interventions will be delivered alongside the NDA to primary care networks (small groups of general practices) across England. Our primary outcome will be HbA1c level at 16-months post-randomisation in young adults with type 2 diabetes and baseline HbA1c ≥ 58 mmol/mol. Secondary outcomes assess the proportion with an HbA1c below recommended thresholds, prescription consistent with recommendations and delivery of recommended care processes. We will investigate impacts on equity. We will explore implementation, engagement and fidelity through interviews, observations, documentary analysis and surveys. An economic evaluation will estimate cost-effectiveness and budget impact.

**Discussion:**

Our study embeds a further evaluation within the NDA, strengthening its role as a national diabetes learning health system. Our findings will have implications for intervention providers and funders seeking improvement in care and outcomes, and for our understanding of large-scale implementation strategies.

**Trial registration:**

ISRCTN 52205353 Registered 12 March 2025. https://www.isrctn.com/ISRCTN52205353.

**Supplementary Information:**

The online version contains supplementary material available at 10.1186/s13012-025-01479-8.

Contributions to the literature

- Feedback facilitation can increase the effectiveness of audit and feedback.
- It is not known whether or how to deliver feedback facilitation at scale.
- We will evaluate the effect of two feedback facilitation interventions delivered to primary care networks across England on the achievement of recommended glucose levels. We will investigate impact on equity and cost-effectiveness, and explore implementation and engagement with the interventions.
- Our study embeds a further evaluation within the NDA, strengthening its role as a national diabetes learning health system and generating learning for implementation scientists, intervention providers and funders.

## Background

Early onset type 2 diabetes, defined as before age 40 years, differs from later onset and is associated with greater risk of complications and reduced life expectancy [1]. Over 145,000 young adults (18–39 years) in England have type 2 diabetes.

The global prevalence of early onset diabetes is increasing [2]. In England, type 2 diabetes is now more common in people under 40 than type 1 diabetes and disproportionately affects people from minority ethnic groups and young adults living in deprived areas. For example, 35% of young adults with type 2 diabetes are from the most deprived quintile, which is considerably higher than the 22% of 60–79 year olds in the same quintile. Prevalence of type 2 diabetes in young adults is almost fourfold higher among Asian people and over twofold higher among Black people relative to people of white ethnicity [1].

The English National Institute for Health and Care Excellence (NICE) recommends clinicians tailor treatment according to individualised patient goals and medication use. An HbA1c below 48 mmol/mol is recommended for many people with type 2 diabetes [3]. Where HbA1c is 58 mmol/mol or higher, NICE specifies additional care: reinforcement of advice about diet, lifestyle, adherence to drug treatment and more intensive drug treatment. In England, over 60,000 adults under 40 years have HbA1c levels ≥ 58 mmol/mol [3] putting them at greater risk of retinopathy, nephropathy, neuropathy, cardiovascular disease, sexual health problems and foot disease [1]. These young adults living with type 2 diabetes face challenges engaging with care, such as greater stigma, work or family commitments, or moving area for education [2]. They are also less likely to receive recommended diabetes care processes such as foot checks, blood pressure and cholesterol monitoring or annual clinical reviews to help reduce risk of complications [1, 4]. Addressing modifiable factors in healthcare such as ways of working, staff skills, knowledge, attitudes and beliefs may support young adults to engage with and respond to care [5]. NHS England (NHSE) has identified care for this patient group as an improvement priority [6].

A range of programmes exist to improve care and outcomes for people with diabetes. Amongst these, the National Diabetes Audit (NDA) aims to drive improvements in care and outcomes. The NDA is a registry, and feedback provider, integrating patient-level primary and secondary care records on people diagnosed with diabetes, with near-universal coverage. Funded by NHSE, the NDA is one of around 60 national audits in England [7]. The NDA provides feedback on recommended processes of care (e.g. number and proportion of people who have their blood glucose levels checked) and attainment of treatment goals (e.g. glucose control) to general practices and primary care networks (PCNs). PCNs are small groups of general practices, typically located close to each other, working together to coordinate care delivery. The median number of young adults with type 2 diabetes and HbA1c above or equal to 58 mmol/mol is 56 (inter-quartile range 3 to 89 young adults). Feedback highlights areas for improvement to stimulate change. Systematic review evidence indicates that audit and feedback modestly improve care delivery [8]. However, adding feedback facilitation as a co-intervention may result in greater improvement [9, 10].

We have developed two feedback facilitation interventions suitable for delivery at scale within the NDA to all PCNs in England: medium-intensity feedback facilitation involving virtual workshops; and low-intensity feedback facilitation delivered through virtual educational materials and email.

Whilst NHSE has agreed to fund the medium-intensity co-intervention, it is unknown whether it is effective or cost-effective and whether such intensity is needed [11]. Embedding the research within the NDA provides resources for delivery, closely reflects usual delivery of such interventions and supports PCN engagement. It also ensures a direct route for implementation of research findings by the NDA as well as offering transferable evidence for other national audits.

We will evaluate the effectiveness of NDA feedback with (i) medium-intensity facilitation to PCNs and (ii) low-intensity facilitation to PCNs compared to NDA feedback alone in reducing the HbA1c of young adults with type 2 diabetes and HbA1c ≥ 58 mmol/mol. We will explore facilitation implementation, engagement, fidelity and tailoring of actions and estimate the cost-effectiveness of medium- and low-intensity facilitation.

## Methods

### Study design

An efficient 3-arm cluster randomised trial using routine NDA data with parallel process and economic evaluations. The facilitation interventions will target PCNs to help them achieve blood glucose goals in young adults with type 2 diabetes. The intervention programme theory and content is described below, and in an adapted Template for Intervention Description and Replication (TIDieR) [12] (Appendix A) and a logic model (Appendix B). The interventions draw upon organisational readiness for change [13] and Normalisation Process Theory [14], supporting participants to commit to change, select tailored improvement actions guided by an informational assessment and to reflexively monitor their collective action. The logic model describes consistency with Clinical Performance Feedback Intervention Theory (CP-FIT) [9].

### Study setting

PCNs in England.

### Cluster eligibility

All PCNs in England.

### Patient eligibility

All young adults (18–39 years) living with type 2 diabetes and an HbA1c level above or equal to 58 mmol/mol in England in the previous year, identified through the national audit before the intervention period (baseline).

### Randomisation

**Fig. 1:**
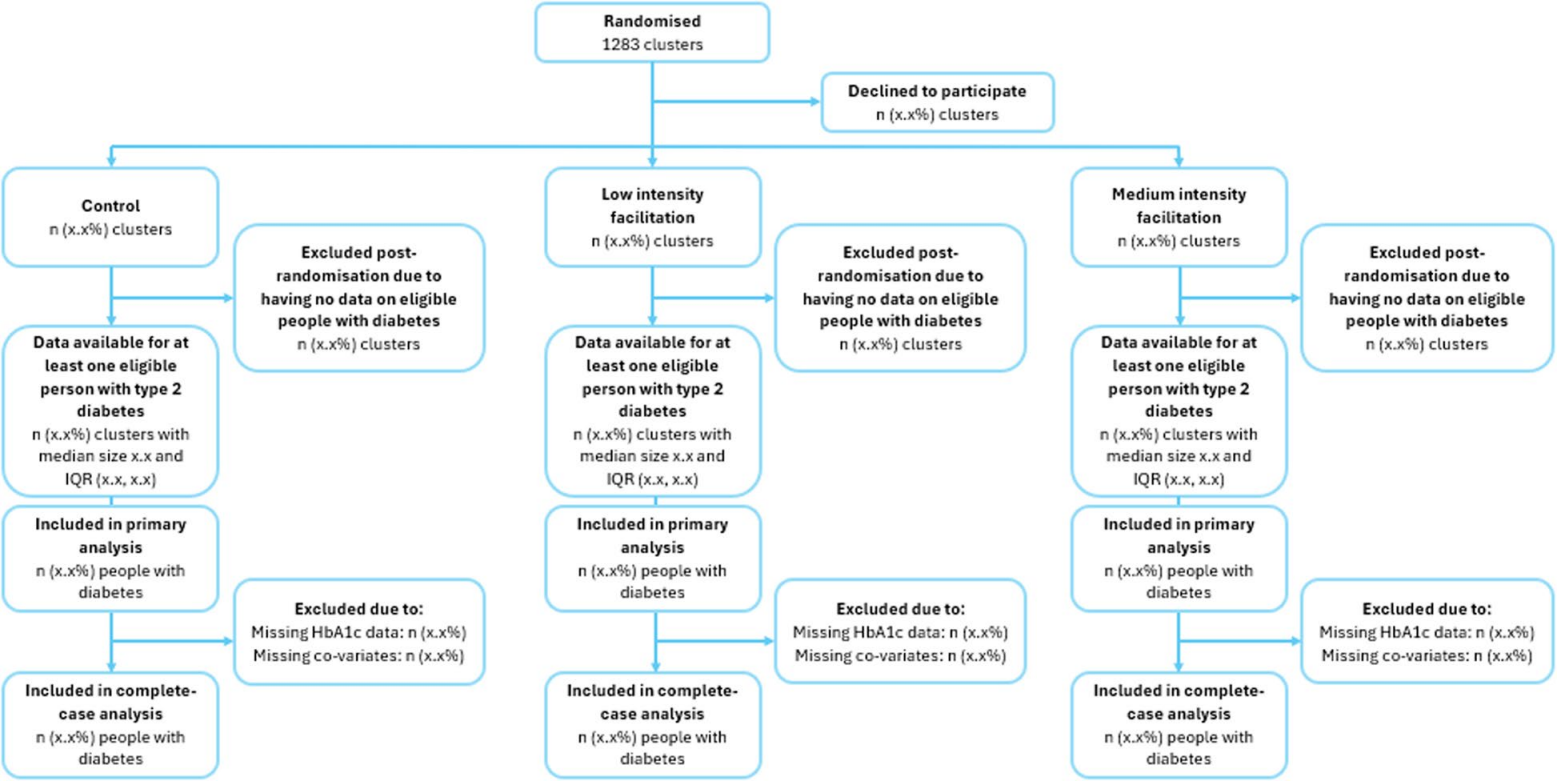
CONSORT flow chart

All PCNs will be randomised to one of three study arms: (i) medium-intensity facilitation with NDA feedback, (ii) low-intensity facilitation with NDA feedback, or (iii) NDA feedback alone (waitlist control group) on a 1:1:1 basis, using a computer-generated minimisation programme within the Clinical Trials Unit (Fig. 1).

### Minimisation factors

We will stratify by PCN-level baseline proportion of young adults with type 2 diabetes who have an HbA1c ≥ 58 mmol/mol (above or below median) and by NHS Integrated Care Board (ICB; NHS organisations responsible for planning and commissioning health services for their local populations). ICBs vary by the number of PCNs (median 27; IQR 19 to 42) and number of young adults with type 2 diabetes and raised HbA1c above 58 mmol/mol (median 1732; IQR 1250 to 2498). Stratifying by ICB will help to minimise any imbalances in patient characteristics including age, sex, ethnicity and deprivation, but we will also account for these patient-level characteristics in the statistical and economic analyses.

### Blinding

Allocation concealment is not feasible in this trial.

### Intervention

Both feedback facilitation co-interventions will be delivered by Diabetes UK (a charity that aims to reduce harm from diabetes) on behalf of the NDA. Both co-interventions support the implementation of care practices by primary care staff and patients to achieve NICE-recommended blood glucose targets. The logic model (see Appendix B) represents the programme theory of how we hypothesise these interventions will produce their outcomes. Intervention development involved patient, public, policy-maker and clinician prioritisation of young adults with type 2 diabetes as the focus. The intervention content was created through a stakeholder workshop involving people with diabetes, policy-makers, clinicians, Diabetes UK employees and implementation scientists, and discussions with an advisory group constituted of people with diabetes. This included consideration of the evidence-based practices and how to implement these practices. It led to the incorporation of content from both young adults describing their experience of diabetes and care, and of vignettes from higher-performing primary care networks.

For both interventions, we propose that the identified evidence-based [15] improvement practices (case management; patient education; promotion of self-management; patient reminders; enhanced prescribing) will improve blood glucose. These evidence-based practices will be more likely to happen if clinical leaders within PCNs undertake collective actions that address local contexts (e.g. to change staff knowledge, beliefs, ways of working). These actions result from setting new behaviour and outcome goals, informed by an informational appraisal and supported by organisational commitment [13]. As shown in the logic model, behaviour change techniques (e.g. credible source, information on consequences [16]) have been linked within both interventions to both informational appraisal and change commitment. Ongoing review of NDA feedback supports participants’ reflexive monitoring of the collective actions taken to improve outcomes.

Intervention components draw on CP-FIT theory [9] hypotheses (e.g. perceived to support positive change rather than punish suboptimal performance; compare recipients’ current performance to that of other health professionals, organisations or regions; provide solutions to suboptimal performance or support recipients to do so).

The low- and medium-intensity feedback facilitation interventions include overlapping behaviour change techniques but differ in terms of resource implications for both healthcare deliverers and PCN recipients. Where content is only delivered in the medium-intensity feedback facilitation (e.g. group delivery of content), this is depicted in the logic model (Appendix B).

PCNs will be offered the interventions to which they have been allocated via an email distributed from Diabetes UK via NHSE regions and ICBs. Staff in PCNs allocated to the medium intensity intervention will be invited to participate in 7 virtual workshops. Staff in PCNs allocated to the low intensity intervention will be offered access to asynchronous web-based training with email support. For each intervention arm, the emails inviting participation will be signed by the clinical and improvement lead and detail that the support for improvement is free, delivered by DiabetesUK and provides learning from higher performing PCNs, learning from young adults, help to identify high-risk population and best practice guidance. The invite will also describe the reason for focussing on young adults with type 2 diabetes and how the learning may help different goals, including improved outcomes, increased efficiency of care, evidence of quality improvement activity and may increase practice income. It will detail the target audience and invite them to register via a virtual registration form.

Waitlist control will receive usual NDA feedback alone. After the trial follow-up period, the PCNs allocated to this group will also receive feedback facilitation. The design will be informed by early process evaluation findings; the NDA contract requires delivery before trial data analysis is possible.

### Outcomes

HbA1c level drawn from routine NDA data at 16-months post-randomisation in young adults with type 2 diabetes and baseline HbA1c ≥ 58 mmol/mol. We have chosen a 16-month follow-up period as it allows for a latent period for (i) PCNs and their member general practices to engage with facilitation, plan and initiate changes in clinical practice and (ii) for patients to consult, consider and start any new treatments or make any suggested lifestyle changes and for any benefits to be reflected in subsequent HbA1c measurements.

Secondary outcomes will comprise:

- Proportion with an HbA1c of < 58 mmol/mol.
- Proportion with an HbA1c of < 48 mmol/mol.
- Proportion prescribed both metformin and SGLT2 inhibitor.
- Proportion in whom the last blood pressure reading (measured in the preceding 12 months) is 140/80 mmHg or less (or equivalent home blood pressure reading).
- Proportion with statins prescribed.
- Proportion receiving NICE-recommended care processes in the preceding 12 months: blood pressure check; serum creatinine testing; urinary albumin: creatinine ratio testing; foot examination and risk classification; smoking status; retinal screening; body mass index; and referral to a structured education programme.

In young adults with baseline HbA1c ≥ 48 and < 58 measurement, we will examine the proportion with HbA1c ≥ 58 mmol/mol at follow-up to check for any unintended distributional consequences from focusing resources on those patients with higher HbA1c at baseline.

### Study procedure

Each cluster will be a PCN, as identified by the NDA. We plan to randomise all 1283 PCNs to one of two interventions or a waitlist control.

Totals of 1150 PCNs and 41,365 patients and an assumed intra-cluster correlation (ICC) of 0.01 and coefficient of variation of 1.62 would provide greater than 99% power to detect a 2 mmol/mol difference in HbA1c between the intervention and waitlist control arm assuming a standard deviation of 18.7 and 10% losses to follow up [17]. This uses the Bonferroni correction to adjust for multiple comparisons (testing two interventions separately against the same control group) with a family-wise type I error rate of 5% (i.e. a 2.5% critical threshold for each comparison).

Estimates for the clustering effects, cluster size and standard deviation are based on the most recent NDA data available from 2021–22. Whilst such reductions in HbA1c are modest at the individual level, they can translate into significant population-level benefits and are comparable to effects by other clinical interventions [18, 19].

### Recruitment

NHS England has 42 ICBs, within which PCNs are nested. NHS England, which commissions the NDA, has a list of ICBs, ICB Clinical Leads and corresponding PCNs. These Clinical Leads include leads for diabetes care, medicines optimisation and practice nurse development. In turn, these ICB Clinical Leads have contact details for the PCNs within their ICB.

Based upon random allocation at the PCN level, ICB leads will be asked to email all PCNs with different messages depending on random allocation to the three groups.

In this way, all PCNs will receive an invite to be involved in work to improve the quality of care. They will not all receive this support at the same time. This is usual practice for the NDA.

We will add to the usual email by informing recipients that the facilitation is being evaluated. Recipients have the option not to engage with the facilitation. Additionally, medium-intensity arm PCNs will be told that this will include observation of what is delivered in the virtual meetings (but not what attendees do). Medium-intensity facilitation arm recipients have the option to attend unrecorded virtual meetings.

### Data collection

Trial outcomes will be assessed using routinely collected individual patient data which is extracted from GP clinical systems by the NDA via the General Practice Extraction Service. We will use NDA-held demographic data in assessing effects on equity. The NDA validates, monitors and reports on data quality [4]; the NDA data quality report provides support that our selected measures are reliable.

The analysis will include outcome data from 12 months before randomisation to 16 months after randomisation.

### Data monitoring

The quality of patient outcome and demographic data is reported and validated by the NDA. A Project Steering Committee (PSC) will meet at least annually to discuss study progress, adherence to protocol, patient safety, and consider new information. A subcommittee of the PSC will be convened where necessary to monitor safety data.

### Data analysis

We will use all available data from all randomised PCNs, according to a detailed pre-specified plan finalised and agreed by the research team before any analyses are undertaken. We will conduct all analyses on the intent-to-treat (ITT) population, in which all PCNs and patients will be included in the analysis according to the group to which they are randomised, regardless of intervention adherence.

We plan no interim analyses except for assessment of progression criteria. We will conduct a single final analysis after the end of the follow-up period, when fully cleaned data are available from the NDA. We will compare characteristics of patients and PCNs lost to follow-up with those not lost to follow-up to assess for attrition bias. A cluster CONSORT diagram will depict the flow of PCNs and patients through the study.

The primary ITT analysis will compare the primary outcome between trial arms, using mixed effects linear regression, with patients nested within PCNs, and with PCNs treated as a random intercept, adjusting for patient-level and PCN-level covariates (including patient age, sex, ethnicity deprivation and PCN-level stratification factors). Estimated mean differences will be reported with confidence intervals, *p*-values and intra-cluster correlation coefficients. For binary outcomes, mixed effects logistic regression will be used and estimated odds ratios will be reported with confidence intervals, *p*-values and intra-cluster correlation coefficients.

Although we expect the level of missing data to be small, we will investigate patterns of missing data and reasons for missing data. We will compare the proportion of missing data between intervention and waitlist control groups. We will build a multiple imputation model assuming data is missing at random for the primary outcome.

Planned subgroup analyses will explore potential moderators of primary outcome treatment effect using key baseline factors (ethnicity, sex, age, and deprivation). This will indicate whether the feedback facilitation contributes towards reducing inequalities in care. Subgroup analyses are exploratory, providing estimates of the direction and size of any interactions.

### Process evaluation

Our theory-informed, integrated process evaluation will involve semi-structured interviews, documentary analysis, survey data collection and observations, with component study level analysis and synthesis.

Guided by the Medical Research Council Framework for developing and evaluating complex interventions [20], we will:

- Describe how the targeted teams and individuals engage with each intervention to support improvement activity and how context influences this work.
- Assess fidelity of design, delivery, receipt and enactment of the interventions.
- Describe how teams enact tailoring.

#### Theoretical approach

We address criticisms that feedback facilitation interventions are ill-defined [11] by illustrating intervention content in a logic model (Appendix B). The process evaluation will draw upon the intervention programme theory. The interventions were developed through co-design with stakeholders and drawing upon evidence and theory [9, 13, 14]. The interventions have defined content including specified behaviour change techniques [16]. The logic model describes the mechanisms through which these active ingredients stimulate collective actions tailored to the local context to generate important outcomes. As such, the design incorporates mechanisms targeting different levels: individual (behavioural), collective (team working) and organisational. The process evaluation will explore whether hypothesised mechanisms for achieving change throughout professional behaviour (what participants do), care delivery (processes of care) and patient-level outcomes (blood glucose) are evident when the interventions are used in practice, and what wider factors affect these mechanism-outcome relationships.

We will use Normalisation Process Theory (NPT) to conceptualise the dynamic processes (coherence, cognitive participation, collective action and reflexive monitoring) during implementation [14]. We will investigate fidelity [21, 22] to assess the extent to which intervention active ingredients are delivered by facilitation deliverers and received and enacted as intended by participants. We will explore contamination between study arms. We will explore whether and how teams tailor their response to local contexts. This will go beyond the fidelity question of whether participants use the tailoring frameworks within the interventions by exploring how teams select strategies, who is involved in this work and when plans are reassessed.

Our analysis will be ongoing and iterative and draw upon other approaches from implementation science where relevant.

#### Participants

We will include healthcare professionals within PCNs (e.g. clinical pharmacists, GPs, practice nurses, managers) and people involved in intervention delivery (e.g. NDA improvement lead, NDA administrator).

#### Sampling and recruitment

For up to 60 process evaluation PCNs: It is not possible for PCNs to give permission for the study as they are not legal entities. Instead, we will seek permission for the study from sampled practices with a local information pack. We will use strategic sampling within the intervention arms (e.g. by baseline performance) aiming to ensure diversity of teams, settings and population characteristics. Sampling considerations relating to specific data collection activities are described below. We will sample from teams who have and have not registered for the intervention. To limit participant burden, we will sample around 20 teams from each arm. We will give all potential participants information about the study and ask interviewees for written informed consent.

All registering PCNs: Given the scale of study participation and relatively small proportion of PCNs that can be included in the in-depth qualitative work, a brief arm-specific online survey will be sent to all PCNs that have registered for one of the interventions.

Documents (e.g. intervention materials) will be analysed as part of the fidelity assessment. The intervention provider will be asked to share data describing attendance and engagement.

Observations will only happen in the medium-intensity arm where, with informed consent, the virtual workshop deliverers will be observed asynchronously to assess fidelity of delivery using recordings routinely made by the NDA. Recipients will be given a choice to attend virtual workshops which are not observed. We will seek to observe up to 50%; participants will be given information about the study and given the choice to attend virtual workshops which are not observed.

#### Data collection

We will use theory-informed, semi-structured interviews. The data will be used for all three process evaluation objectives and to collect health economics data. In the medium intensity arm: up to 20 interviews after the initial virtual workshop, 10 for implementation, engagement and fidelity and 10 for tailoring, and the same post-intervention; in the low-intensity arm, up to 20, post-intervention; in the waitlist control arm, up to 20 interviews to explore ‘implementation as usual’ and contamination (e.g. receipt and use of any materials shared with intervention arm participants). We will interview approximately 3 intervention deliverers to explore fidelity and gather health economics data. We will analyse approximately 28 h of observations.

The observations will be asynchronous using routine recordings made by the NDA and involve a structured analysis of about 50% of the virtual workshops (28 h) from the medium-intensity facilitation. Observation of delivery will inform assessment of fidelity; Data will also be collected to understand tailoring and cost, to supplement and stimulate interview questions. We will not observe low-intensity or waitlist control arms given higher risk of unintended co-intervention effects.

Both the medium- and low-intensity facilitation ask participants to identify influences upon performance and select strategy(s) to address these influences. We will seek and analyse documents such as action plans to explore fidelity and tailoring. We will seek related documents from waitlist control arm participants to understand ‘implementation as usual’.

To supplement interview data, we will seek permission from the Health Research Authority (HRA) for a survey distributed via Diabetes UK to people who registered for one of the interventions. The brief survey aims to gather structured data about tailoring, fidelity, contamination and cost (described in time, grade and any equipment costs). Data will be used for both the process and economic evaluation, to facilitate comparisons between arms and to help reflect the large number of sites not included in the interviews.

#### Data management and analysis

With written, informed consent, we will audio-record and transcribe verbatim all interview data prior to using framework analysis [23] for each process evaluation objective. Data and audit trail will be managed through NVivo. Sykes is involved in both facilitation delivery and process evaluation. To support trustworthiness within the process evaluation, we will explicitly consider critical distance associated with the chief investigator’s (Sykes) contribution to the protocol and to data interpretation. To maintain critical distance, Sykes will not be involved in the fidelity sampling or assessment, as implemented successfully in an earlier study [24].

Analysis will be both inductive and deductive. The assessment of of fidelity and tailoring will largely be deductive, whilst implementation and engagement will primarily be inductive.

Data will be extracted from documents by two researchers for each of the three process evaluation objectives. The extracted data will also inform the later interview topic guides for specific sites. For example, the description of stakeholder engagement and tailoring might stimulate questions exploring the rationale for the proposed approach and/or the extent to which the plans were enacted as intended.

Analysis of fidelity of delivery will be in accordance with recommendations [21]: 80 to 100% adherence to intervention specifications represents ‘high’ fidelity of delivery, 51 to 79% represents ‘moderate’ fidelity, and < 50% or less represents ‘low’ fidelity.

Survey data will be analysed using descriptive statistics (fidelity, cost) and framework analysis (tailoring).

The analysis will be concurrent across each process evaluation objective, to enable earlier findings to inform later data collection and analysis. Interim analyses will be shared with stakeholders (including Experts by Experience, clinicians, policymakers and implementation scientists) to identify avenues for exploration within the data.

#### Integrative analysis

Consistent with our earlier work [24], we will seek to integrate findings between:

i) Data sources within the process evaluation through common data management and coding.
ii) Process evaluation objectives. For example, what the approach to tailoring tells us about fidelity of enactment and engagement with the intervention.
iii) Component studies, through integrative workshops to understand what the findings from each mean to the others and to identify avenues for exploration. For example, if our trial identifies differential effect based upon deprivation, the process evaluation could explore the inclusion of deprivation in identification of actions by PCNs.

Integration will happen through team meetings and integrative workshops involving project team and wider stakeholders, as appropriate.

### Economic evaluation

An analysis plan will be written following Bristol Health Economics Analysis Plan (HEAP) guidance [25] and, as far as possible, the evaluation will align with the trial statistical analysis plan and adhere to the NICE reference case. All analyses will adopt the NHSE perspective and all cost and QALY measures will be discounted at the rate of 3.5% per annum. Our analysis will comprise three components:

1. Within-trial economic evaluation and budget impact analyses.
  Design: An intention-to-treat, cost-effectiveness and cost-consequence analysis with a time horizon of 18 months and budget impact analysis using patient-level NDA data and intervention cost data.
  Data collection: Within-trial biomarker and resource use data collected through the NDA between baseline and 18 months post-randomisation will be used to assess health outcomes and healthcare costs. Healthcare cost data will include primary care prescriptions (glucose lowering drugs, antihypertensives and statins), inpatient (diagnosis, procedure, length of stay) and some outpatient data. The incremental costs of the two interventions when compared to NDA feedback alone will be assessed using a bottom-up, micro-costing approach [26]. This will draw on resource use data collected from people involved in facilitation delivery (e.g. NDA improvement lead, NDA administrator) as a fully integrated component within the interviews and surveys conducted as part of the process evaluation. These resources are likely to span intervention refinement, delivery and response activities; consumable costs incurred (e.g. printed material) and staff time (and grade) required; and additional activities that result from the intervention (e.g. meetings with stakeholders, local team training, additional consultations with patients).
  Analysis: The within-trial cost-effectiveness analysis will assess cost per unit change in HbA1c. The cost-consequence analysis will assess changes in all other NDA biomarker outcomes (e.g. cholesterol). Each resource use item, including staff time, will be costed using unit cost data available from routine sources, e.g. national healthcare databases such as Personal Social Services Research Unit (PSSRU) data [27, 28]. We will seek to capture the variance in costs that might occur across centres and incorporate this uncertainty in the analysis. We will also empirically estimate the denominator sample for deriving the per patient intervention cost. Given the high incidence and prevalence of type 2 diabetes in young adults, and thus potentially large ongoing costs to NHSE of delivering the intervention, an NHSE-wide budget impact assessment will also be conducted. Cost and outcome data will also be analysed for equity relevant subgroups as defined in the trial statistical analysis to inform the equity informative cost-effectiveness analysis (see below).
2. Lifetime cost-utility analysis:
  Design: An intention-to-treat, cost-utility analysis with a time horizon of a lifetime, using intervention cost data, patient-level NDA data and the UK Prospective Diabetes Study Outcomes Model 2 (UKPDS-OM2) patient-level simulation model [29].
  Analysis: The patient-level demographic (e.g. age, ethnicity) and biomarker data collected during the within-trial period will be used as inputs to the UKPDS Outcomes Model v2.2 [29], which is a commercially available microsimulation model, pre-built, externally and internally validated, and recently updated by the University of Oxford to project long-term type 2 diabetes outcomes. The model will be used to estimate incremental lifetime healthcare costs and QALYs between the waitlist control and intervention groups by forecasting annually until death the progression of cardiovascular and type 2 diabetes complication risk factors (e.g. cholesterol, HbA1c, heart rate, smoking status, systolic blood pressure, weight, time since diabetes diagnosis) and the occurrence of eight events (e.g. myocardial infarction, ischaemic heart disease, stroke, amputation, blindness, renal failure). The primary outcome measure will be an incremental cost effectiveness ratio (ICER) assessing the aggregate-level incremental cost per QALY. We will also report net (monetary) benefit, which is a rearrangement of the ICER and estimated as: (λ x QALYs) – Costs, where λ is the willingness to pay threshold per health gain (in the case of NICE, £20,000-£30,000 per QALY). Sensitivity analysis will explore assumptions made in the analysis and parameter uncertainty (e.g., around treatment costs). Decision uncertainty will be illustrated using a scatter plot of incremental cost–QALY pairs (UKPDS-OM2 outputs with 1,000 inner loops and 5,000 bootstraps) and the cost-effectiveness acceptability curve (CEAC).
3. Equity informative cost-utility analysis
  Design: A distributional cost-utility analysis will be undertaken to evaluate the equity impact of the interventions on outcomes alongside average cost-effectiveness [30, 31].
  Data collection: Audit data collected throughout intervention delivery will be used to estimate model parameters to be used within the UKPDS-OM2 for equity groups of interest including socio-economic status, gender, and ethnicity. Data collected within the study will be disaggregated by these equity groups and analysed to inform model input data for each group. The UKPDS-OM2 will be used to estimate lifetime costs and outcomes for each equity group which will be combined to create a distribution of costs and outcomes across the population for analysis.
  Analysis: The distribution of costs and outcomes will be analysed according to equity-informative cost-effectiveness analysis principles [32]. The distribution of costs and outcomes in the control group (without the interventions) will be compared with the distribution of costs and outcomes with each intervention. The inequality in each distribution will be calculated (e.g., using absolute and relative measures of inequality) and compared to quantify the impact of each intervention on health inequalities. The overall equity impact will be plotted against the overall cost-effectiveness (results from the lifetime cost-effectiveness analysis) on the health-equity impact plane and any trade-offs between equity impacts and cost-effectiveness will be analysed. Disaggregated estimates of costs and outcomes for different groups will also be presented to demonstrate the extent to which different groups are expected to benefit from the interventions.

### Public involvement

In addition to involvement in intervention development described above, we are grateful for the involvement of young adults with diabetes in both our research team and intervention delivery team. As members of the team, they will review study progress and input into decisions about changes to protocol. Within the process evaluation, they will provide challenge to emergent findings and help synthesise and interpret the results. In addition, we will discuss project updates with people with diabetes through the NDA Experts by experience group. They will be invited to be part of integrative workshops to synthesise and interpret data from the three component studies.

## Study progress

We have gained ethical and Health Research Authority approval. In April 2025, we allocated 1283 primary care networks and emailed all intervention arm emails to via NHS England regions and integrated care boards to PCNs. We have begun delivery of the medium-intensity arm virtual workshops and started to send materials to the low-intensity arm sites.

## Discussion

The number of young adults with type 2 diabetes is increasing globally. There is evidence that they have poorer outcomes than other age groups with type 2 diabetes. Evidence-based care processes and improvement practices, such as case management and the promotion of self-management, can improve the achievement of blood glucose targets and reduce patient risk. There is variation between primary care networks in the achievement of blood glucose targets and the delivery of recommended care processes. Primary care networks receive feedback from the NDA describing the achievement of blood glucose targets and the delivery of recommended care processes. Providing feedback facilitation may increase the effectiveness of audit feedback. It is currently not clear how this might be delivered at scale to primary care networks across England. We will therefore test two interventions, deliverable at scale, that vary in intensity. Our pragmatic cluster randomized controlled trial with theory-informed process economic evaluation allocates all 1283 primary care networks in England to one of two interventions or waitlist control. Delivery to all primary care networks by the usual provider of similar interventions and using routine data demonstrates pragmatism, builds upon our previous embedded trial and takes a further step towards a national diabetes learning health system.

## Conclusion

Young adults with type 2 diabetes have a reduced life expectancy. Care and outcomes may be improved through the implementation of evidence-based practices. Evaluating the delivery of our specified interventions will provide valuable learning to intervention funders and deliverers. If effective, the interventions will improve both patient and service outcomes. If not effective, the evaluation will provide pragmatic lessons that inform future delivery and improve efficiency.

## Supplementary Information

Supplementary Material 1

## Data Availability

Data sharing is not applicable to this article as no datasets were generated or analysed during the current study. The intervention manual is available from the corresponding author (MS).

## Appendix A

TIDieR framework [12] intervention description

**Table:**

Item number		Low-intensity	Medium-intensity
1.	BRIEF NAME Provide the name or a phrase that describes the intervention.	The EQUIPD2 low-intensity feedback facilitation intervention	The EQUIPD2 medium-intensity feedback facilitation intervention
2.	WHY Describe any rationale, theory, or goal of the elements essential to the intervention.	See logic model	See logic model
3.	WHAT Materials: Describe any physical or informational materials used in the intervention, including those provided to participants or used in intervention delivery or in training of intervention providers.	e-Learning package supported by emails. The e-Learning package will be made available after the intervention period.	Powerpoint slides supported by emails. The slides will be made available after the intervention period.
		Both interventions contain further materials: Searches to identify patients, best practice guidance, checklists, recordings from young adults, videos from high-performing PCNs and links to publicly available web-resources.	
4.	WHAT Procedures: Describe each of the procedures, activities, and/or processes used in the intervention, including any enabling or support activities.	We will ask NHS regions to email PCNs, describing the intervention and providing a registration link. Registrants will be sent access to the e-Learning package or the virtual workshop invites.	
		e-Learning registrants will be able to opt-in to approximately 2-monthly emails intended to share learning and respond to both clinical and quality improvement questions. The responses will be general guidance not specific to patients or sites respectively.	Virtual workshop registrants will be sent diary invites for the virtual workshops. They will be given information about which virtual workshops are not recorded. They will select the dates for the didactic virtual workshops in advance. For the 5 interactive virtual workshops, they will receive invites for all virtual workshops and be asked to choose which they attend.
5.	WHO PROVIDED For each category of intervention provider (e.g. psychologist, nursing assistant), describe their expertise, background and any specific training given.	The intervention providers across both interventions are clinical experts, quality improvement leads. In addition, both interventions include asynchronous content provided by young adults with type 2 diabetes and clinical and non-clinical leads at high-performing PCNs.	
6.	HOW Describe the modes of delivery (e.g. face-to-face or by some other mechanism, such as internet or telephone) of the intervention and whether it was provided individually or in a group.	Via internet to the individual	Via MSTeams to multi-site groups. Each will have multiple date options. We will report on the size of these groups, which may vary based upon the dates participants choose to attend.
7.	WHERE Describe the type(s) of location(s) where the intervention occurred, including any necessary infrastructure or relevant features.	Internet	MSTeams
8.	WHEN and HOW MUCH Describe the number of times the intervention was delivered and over what period of time including the number of sessions, their schedule, and their duration, intensity or dose.	Sites will have access to 16 months. The eLearning takes approximately 90 mins to complete.	Sites will have access to 16 months. There will be 7 virtual workshops, two are more didactic, and 5 planned to be interactive.
9.	TAILORING If the intervention was planned to be personalised, titrated or adapted, then describe what, why, when, and how.	The interventions will not be tailored, but are intended to support local tailoring to identify influences and select actions that reflect local context.	

## Appendix B

Logic model for the medium- and low-intensity interventions

## Abbreviations

EQUIPD2: Evaluation of quality improvement for people with type 2 diabetes
HbA1c: Haemoglobin A1c (a measure of glucose control)
HRA: Health Research Authority
ICB: Integrated Care Board
ICER: Incremental cost-effectiveness ratios
ISRCTN: International Standard Randomised Controlled Trial Number
ITT: Intention to treat
λ: Willingness to pay threshold per health gain
NDA: National Diabetes Audit
NHSE: NHS England
NICE: National Institute for Health and Care Excellence
PCN: Primary Care Network
PSC: Project Steering Committee
QALY: Quality adjusted life years
TIDieR: Template for Intervention Description and Replication
UKPDS-OM2: UK Prospective Diabetes Study Outcomes Model 2
